# Experiences of Accreditation of Medical Education in Taiwan

**DOI:** 10.3352/jeehp.2009.6.2

**Published:** 2009-12-20

**Authors:** Chi-Wan Lai

**Affiliations:** CEO, Taiwan Medical Accreditation Council, Taipei, Taiwan.

**Keywords:** Accreditation, Medical Education, Taiwan

## Abstract

This review aims to introduce the Taiwanese Medical Accreditation System: its history, role and future goals. In 1999, the Ministry of Education, Taiwanese Government commissioned the non-profit National Health Research Institutes (NHRI) to develop a new medical accreditation system. According to that policy, the Taiwan Medical Accreditation Council (TMAC) was established in the same year. The council serves a similar function to that of the Liaison Committee on Medical Education (LCME) of the United States and the Australian Medical Council (AMC). The accreditation process consists of a self-assessment plus a four-day site visit by a team of eight medical educators that are headed by one of the council members of the TMAC. The first cycle of initial visits was completed from 2001 to 2004. Subsequent follow-up visits were arranged according to the results of the survey with smaller-sized teams and shorter periods. There is evidence to suggest that the majority (seven of eleven) of the medical schools in Taiwan have made good progress. TMAC's next step will be to monitor the progress and raise the standard of medical education in individual schools with a homogenous, superior standard of medical education.

## INTRODUCTION

In this article, I would like to share Taiwan's experiences in the accreditation of medical education. I shall first briefly describe the education system in Taiwan before I talk about the history of accreditation of medical education. I shall next explain the role of the Taiwan Medical Accreditation Council (TMAC) [1] and end with an outline of our future goals.

## EDUCATIONAL SYSTEM IN TAIWAN

The first medical school in Taiwan was established in 1897 under the Japanese regime, and now there are a total of twelve medical schools-four are public (national), and eight private in the country. One of the private schools was just founded this (2009) year, and has yet to go through the accreditation process. All medical schools accept students who are high school graduates for a seven-year curriculum (high school leaver system) that includes a one-year internship. Between 1982 and 1991, five medical schools tried a "post-graduate entry" (post-baccalaureate) system in addition to the regular high school leaver system, but only one school has continued with such a "dual" entry system since 1992. Every year, 1,300 students have graduated from medical schools. The population of Taiwan is 23,000,000 and the number of physicians is 35,000 so that there are 15 physicians per 10,000 people.

The seven-year curriculum in medical schools has been basically divided into three parts: pre-medical education for the first two years, followed by basic sciences for two years, and clinical medicine (including a clerkship and internship) for three years. In the last few years, there have been efforts to put an emphasis on medical humanities and general studies in the pre-med years, and schools are discouraged from offering biomedical sciences courses during that period. Nonetheless, we do encourage early exposure to patient contact during pre-med in order to raise the students' sensitivity to human suffering. There is also a movement towards more integration of basic and clinical sciences in the subsequent four years. Many medical schools have shown interest in innovative teaching, learning and assessment methods, and have been practicing small group teaching, problem-based learning (PBL), Objective Structured Clinical Examination (OSCE), etc. A brief comparison of the Korean and Taiwanese medical education systems is summarized in Table 1.

## HISTORY OF ACCREDITATION OF MEDICAL EDUCATION

The collegiate accreditation system in Taiwan was launched in 1975 when the Ministry of Education (MOE) started using separate accreditation systems for various schools and departments. However, there was never an independent accreditation organization devoted to medical education until 1999, when the Taiwan TMAC was established.

The birth of the TMAC was partly related to the impact of the 1998 study conducted by the National Committee on Foreign Medical Education and Accreditation (NCFMEA), under the auspices of the US Department of Education to review the standards used by different countries to accredit their medical schools. In that report, medical education in Taiwan was deemed as "non-comparable" to the US system on several accounts, and the lack of an independent accreditation organization for medical education was considered a major problem. This subsequently set off a series of meetings among medical educators in the country, in which I had the honor of participating. We all recognized that at a time when calls for reform in the educational and judicial systems were strong in Taiwan, criticism from a foreign country of our medical education indeed served as a momentum for a timely overhaul of the medical accreditation system. Furthermore, we all agreed that the main function of medical schools is to provide decent medical education to their students, the quality of which has an important impact on the health of the people. Medical education is a very special kind of education, often referred to as "professional education" and the accreditation of medical schools in countries such as the United States, Canada, Great Britain, and Australia, has been conducted independently with standards often surpassing those of other forms of higher education. To bring the somewhat archaic local medical education accreditation system up-to-date, the MOE then commissioned the non-profit National Health Research Institutes (NHRI) to develop a new medical accreditation system in the spring of 1999. After some rigorous planning, an independent TMAC was established by the NHRI under the leadership of Dr. Kun-Yen Huang, M.D., Ph.D., founding dean of the National Cheng-Kung University Medical College. The council serves a similar function to that of the Liaison Committee on Medical Education (LCME) of the United States and the Australian Medical Council (AMC).

## ORGANIZATION OF THE TAIWAN MEDICAL ACCREDITATION COUNCIL

The TMAC's membership consists of eleven representatives chosen from outstanding medical educators who are faculty members of medical institutions both within and outside Taiwan. The council's Selection Guidelines for Membership clearly define the potential candidate pool: 1) senior faculty with a proven record of longstanding dedication to the education of medical students; 2) senior faculty whose expertise is in education or educational psychology; 3) scholar/educators in liberal arts education; and 4) senior physicians outside of medical educational institutions whose views reflect societal needs. A nominating committee to select the membership of the TMAC is comprised of the chairman and five senior members who review nominations by the MOE, NHRI (which subsequently has been changed to the Higher Education Evaluation and Accreditation Council of Taiwan), and Medical School Deans' Council together.

## PROCESS OF THE SITE VISITS

The accreditation process consists of a self-assessment plus a four-day site visit by a team of eight medical educators with backgrounds in basic science, clinical science and liberal arts that are headed by one of the council members of the TMAC. It should be pointed out that for the TMAC's initial visit, there are always two team members who are medical educators from abroad with a Taiwanese background.

The process of the assessment visit is as follows: 1) Examiners are given a month's time to review the self-assessment package of the school undergoing the accreditation; 2) An exchange forum should be arranged among examiners every evening during the on-site evaluation; 3) The assessment team reserves the right to attend any lecture, conference or bed-side teaching and interview any student, faculty or staff member, or go anywhere on campus or in the hospital; 4) The leader should sort and compile the findings shortly after the on-site evaluation and submit the complete report to the council within three weeks. The report should include a concrete listing of the merits and shortcomings of the school in order to facilitate the final ruling by the TMAC.

The categories and items reviewed during the site visit include the following areas:

1. Administration and resources
  Teaching
  general education, including humanities and ethics basic medical education
    clinical education, including teaching hospitals
  Research
  Assessment of student affairs and guidance about collecting student evaluations

After the review, there are suggestions for the site school. After returning from the site visit and review, council members meet to audit the report by the on-site evaluation team to develop the final conclusion and submit that to the MOE. The assessment resolutions are in three categories: Accredited (full or conditional); on probation; non-accredited. Meanwhile, the accreditation results will become public through the MOE website. Accreditation, however, will not rank schools. The results provide guidance for newly established schools.

The process started in 2001, and the first cycle of initial visits was completed in 2004. Subsequent follow-up visits were arranged according to the results of the survey with smaller-sized teams and shorter periods. A full review of the results of the initial and follow-up visits was conducted by the TMAC in subsequent years, and up to the last survey in 2007, there is evidence to suggest that the majority (seven of eleven) of the medical schools in Taiwan have made good progress with defining their own missions and objectives, and have raised their standards of education sufficiently to meet the approval of the TMAC. The remaining four schools will still require assistance for further improvement before they can be accepted under the TMAC's current guidelines. Of the four, three are "accredited (conditional)", while one is on "probation".

## FUTURE GOALS OF THE TAIWAN MEDICAL ACCREDITATION COUNCIL

From its inception in 1999 to the present, the TMAC has accomplished its mandate as the sole accreditation council for the eleven (recently increased to twelve) existing medical schools in Taiwan. It was reviewed and deemed "comparable" by the NCFMEA in 2002 and continues to maintain such a status in the recent "re-determination" in March 2009.

Looking forward, the TMAC's role will be to further motivate all medical schools in Taiwan towards continuous improvement in the deficiencies described in the detailed accreditation report of each school. In this endeavor, TMAC's main emphasis, however, will focus on aspects of reform, including faculty development, selection of medical students, upgrading all twelve medical schools to more equal standards, and governance and collaboration.

## CONCLUSION

Ten years after the TMAC was first established, its mission has developed from mere accreditation to assistance in upgrading the level of education for medical schools in Taiwan. Now that the weaknesses of these schools have been identified, the TMAC's next step will be to monitor the progress and raise the standard of medical education in individual schools, with the ultimate goal of achieving a homogenous, superior standard of medical education for all schools in Taiwan. TMAC is also in the process of revising standards of accreditation and standardizing the process of site visits. I hope that our concerted efforts can continue to improve the quality of medical education in Taiwan.

## Figures and Tables

**Table 1:**
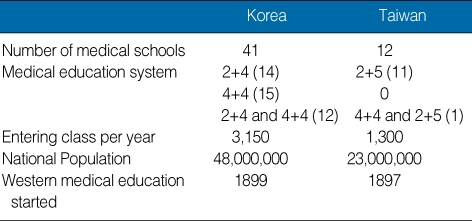
Brief comparison of the Korean and Taiwanese medical education systems
